# An observational study of an adjusted patient blood management protocol intended to lower rates of transfusion following total knee arthroplasty in patients with preoperative anemia

**DOI:** 10.1186/s13018-023-04404-1

**Published:** 2023-12-02

**Authors:** Hervé Hourlier, Peter Fennema

**Affiliations:** 1 Department of Orthopaedic Surgery, Polyclinique de La Thiérache, Rue du Dr Koral, 59212 Wignehies, France; 2 Centre Hospitalier de Fourmies, 59610 Fourmies, France; 3 AMR Advanced Medical Research GmbH, Männedorf, Switzerland

**Keywords:** Patient blood management, Tranexamic acid, Total knee arthroplasty, Blood transfusion, Anemia, Epoetin alfa

## Abstract

**Background:**

Patients with preoperative anemia have a higher risk of requiring blood transfusion after major orthopedic surgery due to increased blood loss and closer transfusion thresholds. Various patient blood management (PBM) policies aim to reduce transfusion rates. This observational study aimed to investigate blood loss and evaluate the effectiveness of an adjusted surgical PBM protocol in patients with anemic chronic disease (ACD) undergoing elective total knee arthroplasty (TKA).

**Methods:**

A consecutive cohort of patients underwent elective unilateral TKA with an adjusted PBM protocol. The protocol consisted of epoetin (EPO) alfa therapy prescribed by the surgeon, routine administration of tranexamic acid (TXA), and standardized postoperative pharmacologic prophylaxis for thromboembolism. The performance of this PBM protocol was analyzed in patients with a baseline hemoglobin level of less than 12 g/dl. Hemoglobin levels were controlled at admission, on postoperative day (POD) 1, and on POD 7 ± 1. A bleeding index (BI-7) was used as an estimate of blood loss up until POD 7. Multiple linear regression was used to assess whether there were any differences in BI-7 between ACD– and ACD + patients.

**Results:**

A total of 751 patients with complete hemoglobin monitoring were included in the study. Of these patients, 68 (9.1%) had a baseline hemoglobin concentration of less than 12 g/dl (ACD group). In this group, 28 patients (41.2%) received preoperative EPO therapy. The mean adjusted BI-7 for the study population was 3.0 (95% CI, 2.9 to 3.0) g/dl in the ACD– group and 2.3 (95% CI, 2.0–2.6) g/dl in the ACD + group. The difference in BI-7 was statistically significant (difference, 0.6 [95% CI: 0.3 to 0.9] g/dl, *p* < 0.001). No major complications occurred in the ACD + group, whereas there were three complications in the ACD– group (*p* = 1.00).

**Conclusions:**

ACD patients undergoing TKA did not have an increased risk of bleeding or bleeding complications with the use of the adjusted PBM protocol. None of ACD patients required transfusion. ACD patients undergoing TKA experienced significantly less blood loss than patients with no preoperative anemia with the use of the adjusted PBM. We now consider the use of EPO in ACD patients with no nutritional deficiencies undergoing TKA at a baseline hemoglobin level of 11 g/dl. However, this should be validated in larger cohorts with a higher prevalence of ACD patients.

## Introduction

Preoperative anemia affects 7–35% of patients undergoing total knee arthroplasty (TKA) [1]. Its prevalence depends on the definition of anemia. Previous studies have shown that the occurrence of preoperative anemia is an independent risk factor of postoperative transfusion, 30-day readmission, thrombosis, and mortality following major surgery [2–4]. In the context of TKA, transfusion is associated with the risk of surgical site infection, major complications, longer hospital stays, and even mortality [5–7]. Further, transfusion increases hospitalization costs and resource utilization [7, 8].

In 2005, we developed a blood management protocol (PBM) based on the use of drugs instead of autotransfusion for total joint arthroplasty [9]. Following this protocol, tranexamic acid (TXA) was administered routinely, and epoetin alpha (EPO) therapy was prescribed in accordance with the product’s instructions for use (standardized regimen of one weekly preoperative 40,000 IU epoetin alpha injection for 4 weeks) and national guidelines (baseline hemoglobin [Hb] between 10 and 13 g/dl). Five years later, we audited our PBM plan, and some changes were implemented to optimize the cost-effectiveness of EPO [10]. Specifically, EPO therapy was prescribed by the surgeon (instead of an anesthetist), the course of EPO therapy was limited to two preoperative injections (instead of four), the dosage was halved (20.000 IU instead of 40.000 IU), and the upper preoperative baseline Hb threshold for implementing EPO was lowered from 13 g/dl to 12 g/dl for both males and females. In addition, the routine prescription of oral iron supplementation was abandoned in the absence of a specific deficiency. The aim of this adjusted PBM was to achieve an efficient, easily applicable, economical, and safe way to perform TKA with a minimized risk of perioperative transfusion.

Recently, we reported on the effectiveness and safety of this adjusted PBM in a large cohort of patients who underwent elective total hip arthroplasty, including patients with renal impairment who are considered at risk for transfusion during the TJA procedure [11]. Here, we report on the outcomes of this adjusted surgical PBM in patients undergoing primary unilateral TKA with preoperative anemia. Patients with anemic chronic disease (ACD) are usually slightly older and fragile, with cardiovascular and renal morbidities. They often use long-term anticoagulant therapy, exposing them to bleeding complications.

The primary aim of this study was to investigate the transfusion rate of ACD patients during primary TKA. A secondary aim was to compare blood loss in preoperative anemic and non-preoperative anemic patients undergoing TKA surgery. We aimed to determine if ACD patients undergoing TKA with the use of our adjusted PBM would experience less blood loss than patients without preoperative anemia. Such a result would be particularly clinically relevant because it could provide new guidance for implementing preoperative EPO therapy in ACD patients undergoing TKA.

## Materials and methods

### Study design

This is a retrospective study involving all primary elective TKA surgeries performed by the senior author (HH) from January 10, 2013, to January 13, 2023. During that decade, all participants received the same standardized PBM. The inclusion criteria were primary unilateral TKA during the abovementioned period and the availability of laboratory parameters at specific time points: the first preoperative Hb level up to three months before surgery (Hb baseline), the second preoperative Hb level at admission (one day before surgery (POD –1), and the third Hb measurement at 7 ± 2 days following the skin closure (POD 7). The exclusion criteria were revision partial or TKA, bilateral TKA procedures with a second operation interval of less than three months, a septic or oncological etiology procedure, and a recent fracture around the knee. Of 764 consecutive TKA patients, two were excluded because of recent trauma, and another 10 were excluded because of the lack of Hb values at POD 7. Nevertheless, none of the excluded patients had preoperative baseline anemia. Their Hb baseline levels ranged from 13.8 to 17 g/dl, and none were transfused during the first postoperative week. All patients included in this study took part in an adjusted PBM protocol, which included management of preoperative anemia, routine use of TXA, and standardized pharmacologic postoperative thromboembolism prophylaxis.

### Surgical technique and *perioperative* blood management

The senior author (HH) conducted the surgeries for all patients and was responsible for perioperative blood management.

### Preoperative protocol

At the preoperative surgical consultation, patients at risk of being transfused because of low Hb, advanced age, low body weight, female gender, and cardiovascular disease [12, 13] were identified by the surgeon, who prescribed EPO alpha in the absence of contraindications (allergy to epoetin, uncontrolled arterial hypertension) and nutritional anemia.

The EPO prescription included two subcutaneous injections of EPO alpha (Binocrit 20.000 UI, Sandoz AG, Basel, Switzerland) at home once a week for two weeks prior to surgery.

Patients taking anticoagulation medications were instructed to temporarily pause their anticoagulant therapy a few days before the surgery. No preoperative autologous blood donation or pretransfusion was conducted. The day before the surgery, a blood count was performed on all patients.

### Intraoperative PBM protocol

Intraoperatively, a minibag of 150 ml saline 0.9% solution was used to administer a single-shot IV of 20–30 mg/kg TXA before making the skin incision, as per routine [14]. Patients who had a history of venous or arterial thromboembolic events within the previous three months did not receive TXA. The standard antibiotic regimens were given 30–60 min before the skin incision. Apart from two patients, general anesthesia was used, preceded by blocking of the sciatic and femoral nerves guided by ultrasound. Fluid IV management was restrictive. All surgeries were performed manually, and tourniquets were inflated at 250 mm Hg. Tourniquets were only used if no vascular lesions were seen on the arterial Doppler exam conducted at admission. Overall, 45 patients (6.0%) did not receive limb ischemia. A midline medial parapatellar approach was made with an eversion of the patella. The cruciate ligaments were sacrificed. The surgical principles of the measured resections and mechanical alignment were followed. Whiteside’s line was used to set the femoral rotation. A cemented PS TK prosthesis was implanted routinely. No rotating hinge prosthesis was used in this cohort, despite some severe knee deformities. The implant most used in this series was the GMK PS TK (Medacta, Switzerland), which consisted of a monobloc full polyethylene tibial component and a full polyethylene anatomic patellar implant. The surgeon did not administer any analgesic local infiltration since all patients underwent ultrasound-guided nerve blocks. Further, no blood salvage system or deep surgical drain was utilized. A knee compression bandage was applied and maintained for one hour after wound closure.

### Postoperative protocol

Six to eight hours after closure, 10 mg of oral rivaroxaban (Xarelto, Bayer, Lille, France) was given to initiate pharmacological venous thromboembolism prophylaxis, which was prolonged for a minimum of 14 days. The application of another antithrombotic drug was avoided during this period of time [15]. Multimodal pain management and transfusion were performed in collaboration with the anesthesiologist. In patients without comorbidities, blood transfusion was set at an Hb threshold of 7 g/dl. Meanwhile, in patients with anemia symptoms, preexisting cardiac insufficiency, or coronary heart disease, blood transfusion was set at a threshold of 8 g/dl. No patient received EPO during the postoperative period.

On the seventh day before surgery, a blood count was completed at home or in the hospital.

### Data collection

Data on perioperative variables were recorded prospectively by the senior author. In this study, Hb levels were assessed exclusively through venous blood samples analyzed by the same laboratory. Measurements were taken upon admission (postoperative day [POD] –1), on POD 1, and on POD 7 ± 1. For patients discharged before POD 5, a written prescription was given to the patient to complete a blood count at home at POD 7. Any blood transfusions were recorded.

Preoperative variables were patient age, sex, body mass index (BMI), American Society of Anesthesiologists comorbidity scores (ASA), use of antithrombotic, preoperative Hb level, and Modification of Diet in Renal Disease (MDRD). Intraoperative variables were anesthesia type (general or spinal), TXA use, tourniquet use, ischemia duration tourniquet time, and operative time. Postoperative variables were Hb level and transfusion occurrence. Complications were documented for 60 days, during which major complications were recorded, including death, perioperative myocardial infarction, cerebrovascular accident, proximal deep venous thrombosis, bleeding complications, infection, and symptomatic pulmonary embolism. The secondary study endpoint was a blood transfusion.

Patients were considered to have been transfused if they received any allogeneic red blood cell unit from the day before surgery to the seventh postoperative day. The bleeding index on POD 7 (BI-7) was the primary endpoint, which was calculated by adding the drop of Hb in g/dL and the number of RBC units infused during those two time points [16]. In detail, the BI-7 formula was as follows: Hb level (g/dL) at POD –1 minus Hb level at POD 7 plus the number of units of packed red blood cells (PRBCs) transfused between those two time points, based on the assumption that the transfusion of one PRBC increases Hb by 1 g/dl [16, 17].

Written informed consent was obtained from all patients prior to the commencement of the study. None of the patients in this study stated any religious objections to blood transfusions. The study, which was purely observational and did not alter standard clinical practice, was not subject to ethics committee approval according to French law.

### Statistical analysis

The cohort was divided into two groups based on whether the preoperative baseline Hb level was < 12 g/dl (anemia group) or ≥ 12 g/dl (non-anemia group). Descriptive statistics for the categorical variables are reported as frequencies and percentages, and those for continuous variables are reported as means and standard deviations.

Categorical variables were compared between the two groups using Pearson’s chi-square and Fisher’s exact tests. Continuous variables were assessed for differences between the two groups using t-tests with unequal variances. The primary research question was examined using multiple linear regression. Adjustments were made for clinically relevant confounders, including age, sex, BMI, surgical time, use of EPO therapy, and operative time. We present both unadjusted and adjusted estimates, providing insight into the influence of these potential confounders. P values below 0.05 were considered to indicate statistical significance. Statistical analyses were performed using Stata version 15.1 (StataCorp, College Station, TX, USA).

## Results

Out of the cohort of 751 patients, 68 individuals (9.1%) underwent Total Knee Arthroplasty (TKA) with an initial Hb level less than 12 g/dl, comprising the ACD + group, while the remaining 683 patients exhibited a baseline Hb level of 12 g/dl or higher, constituting the ACD- group. The characteristics of the ACD + and ACD– groups are presented in Table 1.

**Table 1:** Baseline characteristics

	ACD- (*n* = 683)	ACD + (*n* = 68)	*p* value
Age [years]	71.9 ± 7.9	75.9 ± 9.3	< 0.001
Sex (Female)^§^	412 (60.4%)	52 (76.5%)	0.009
BMI [kg/m^2^]	32.2 ± 6.1	31.7 ± 6.9	0.539
Hb at the preoperative consultation [g/dl]	14.0 ± 1.2	11.3 ± 0.6	< 0.001
Hb the day before surgery [g/dl]	13.8 ± 1.2	12.0 ± 1.0	< 0.001
Chronic antithrombotic drug use^§^	269 (39.4%)	30 (44.1%)	0.102
Preoperative EPO^§^	1 (0.1%)	28 (41.2%)	< 0.001
TXA use^§^	655 (96.0%)	67 (98.5%)	0.503
ASA^§^			0.001
- ASA 1	82 (12.0%)	1 (1.5%)	
- ASA 2	518 (76.0%)	41 (69.1%)	
- ASA 3	82 (12.0%)	20 (29.4%)	
Mean corpuscular volume [fL]*	93.4 ± 4.9 (81 – 113)	91.7 ± 6.2 (77.7 – 115.5)	0.008
Preoperative MDRD [ml/mn/m^2^]	83.7 ± 21.5	72.3 ± 29.4	0.001
Ischemia duration [min]	50.4 ± 18.2	46.7 ± 17.6	0.113

Presented as mean ± standard deviation, except * presented as mean ± standard deviation (range), ^§^ presented as number of observations (%). Abbreviations: ACD, anemic chronic disease; ASA, American Society of Anesthesiology; EPO, erythropoietin; MDRD, Modification of Diet in Renal Disease; TXA, tranexamic acid

In the ACD + group, 28 patients (41.2%) received preoperative EPO therapy. In the ACD + group, no patients received blood transfusions. In the ACD- group, one patient with a baseline Hb of 12.2 inadvertently received EPO therapy. One patient received a blood transfusion.

The unadjusted BI-7 was 3.0 (95% CI, 28.9 – 30.6) g/dl in the ACD– group and 2.2 (95% CI, 1.9 – 2.4) g/dl in the ACD + group (*p* < 0.001). The mean adjusted BI-7 for the study population was 2.9 (95% CI, 2.9 to 3.0) g/dl in the ACD– group and 2.3 (95% CI, 2.0–2.6) g/dl in the ACD + group. The difference in BI-7 between the ACD + and non-anemic groups was statistically significant (difference, 0.6 [95% CI: 0.3 to 0.9] g/dl, *p* < 0.001) (Table 2). The small difference observed between the unadjusted and adjusted estimates suggests that there was minimal confounding from the control variables. The BI-7 in patients administered TXA was 2.9 (95% CI, 2.8—2.9) g/dl. In contrast, patients who did not receive TXA exhibited a BI-7 of 3.8 (95% CI, 3.4—4.2; *p* < 0.001) g/dl. No major complications occurred in the ACD + group. In the ACD– group, there were three major complications (*p* = 1.00): one patient died from an unknown cause two weeks postoperatively, another patient experienced a pulmonary embolism, and a third patient developed a proximal DVT, both instances occurring one month after surgery, after the discontinuation of anticoagulation.

**Table 2:** Results

	ACD- (*n* = 694)	ACD + (*n* = 58)	*p* value
BI-7	2.9 (95% CI, 2.9 to 3.0)	2.3 (95% CI, 2.0–2.6)	< 0.001
RBC transfusion between day before surgery and POD 7 ^§^	1 (0.1%)	0 (0.0%)	1.00
Hb on POD 7	10.9 (95% CI, 10.8–11.0)	9.9 (95% CI, 9.6–10.1)	< 0.001

Presented as mean (95% confidence interval), except ^§^, presented as number of observations (%). Abbreviations: ACD, anemic chronic disease; BI-7, bleeding index determined on postoperative day 7; Hb, hemoglobin; RBC, red blood cells

## Discussion

Reducing the use of allogenic blood transfusion in TKA is important for improving outcomes, mitigating risks to patients, and lowering healthcare costs [5, 6]. In the last decade, there have been impressive reductions in the rate of blood transfusions during TKA due to the use of TXA and the adoption of restrictive transfusion protocols [7, 18]. Currently, transfusion in elective TKA is uncommon in the absence of preoperative anemia. A low preoperative hematocrit or Hb level is the most important factor in blood transfusion after primary TKA. Baseline Hb level is a preoperative variable that can be modified using iron, folates, vitamin B12, and or EPO, but these pharmacologic agents are underused [19].

### Findings

In the present patient series, no ACD patients received a blood transfusion during the first week. None had bleeding complications or reached the transfusion thresholds during the first perioperative week. To the best of our knowledge, this series is the first to show a blood transfusion rate of zero in a consecutive series of ACD patients undergoing primary TKA surgery. Importantly, cell savers and autotransfusion were not utilized during the study.

The most important finding of this study was that the BI-7 was lower in ACD + patients than in patients with no preoperative anemia. The present study is the first we are aware of to show a significantly lower blood loss in patients with baseline Hb levels < 12 g/dl than in those with baseline levels > 12 g/dl, all treated with the same PBM protocol. This contrasts with the results of a recent multicenter study that showed higher blood loss in preoperative anemic patients than in non-anemic patients, regardless of the use of a PBM [19]. Our finding has important clinical implications: preoperative Hb levels do not need to be highly optimized before TKA in ACD patients due to their low blood loss.

We believe that EPO therapy is indicated prior to the TK procedure in ACD patients without nutritional anemia who have a preoperative baseline Hb < 11 g/dl. Another important point is that a low dose of EPO is sufficient to raise Hb to 11 g/dl. This implies that the use of EPO has become more economical. Another overall positive finding is that optimization of preoperative Hb in contemporary TKA performed with blood conservation is not necessary in the overall population, only in patients with Hb baseline levels < 11 g/dl.

### Blood strategies

Current PBMs are multimodal, with variations in management common but under researched. For example, there is little agreement regarding the best preoperative diagnostic strategy and the use of erythropoiesis-stimulating biosimilar agents as a primary treatment. In addition, no consensus exists regarding the upper threshold level of baseline Hb to use EPO therapy. Pierson suggested using EPO in patients with Hb baseline levels < 12 g/Dl based on a mathematical formula that accounts for a restrictive transfusion threshold (7 g/dl) and a mean drop of 3.8 g/dl Hb (+ 1 SD) in TKA [20]. A few subsequent studies found that 12 g/dl was one point too low and that a threshold of 13 g/dl and above would be more appropriate. Currently, the mean drop in Hb in primary TKA is reduced to 3 g/dl with the use of TXA. However, a transfusion threshold of 8 g/dl remains the rule in patients with cardiovascular disease. Using Pierson’s formula [20] with updated parameters, including a BI-7 of 2.1 ± 1.1 g/dl and a transfusion threshold of 8 g/dl, the upper level for Hb optimization in ACD patients with cardiovascular disease is 11.2 g/dl. In the present series, all but one patient with baseline Hb levels < 11 g/dl received EPO, and none received a blood transfusion the first perioperative week.

### Role of the surgeon in blood strategies

In most hospitals, the risk of transfusion is evaluated at the preoperative anesthetic visit. Patients exposed to transfusion are submitted to an Hb optimization program whenever they are found to be anemic and can comply with the program, which requires time and sometimes postponement of planned surgery. In the present series, no surgery was delayed due to the implementation of EPO therapy, which was prescribed in line with the date of surgery.

### Use of epoetin alfa

The use of EPO in TKA is justified if it reduces complications; otherwise, it exacerbates costs, increase thrombotic risk, and has a carbon consequence [21]. In the present series, EPO was used before the surgical procedure since EPO therapy is considered more efficient when given prior to surgery. The EPO regimen was prescribed parsimoniously by the surgeon with a short course and a low dosage. Specifically, the regimen of two injections studied by Rosencher was employed, with a dose of 20,000 IU per injection [22]. This dose was supported by Feagan, who found no significant difference in the increase of preoperative Hb using either 20,000 IU or 40,000 IU of EPO alpha [23]. Patients receiving EPO were not given additional specific antithrombotic therapy since only a low dose of EPO was administered in a short course. Despite the prescription of a lower-than-average dose of EPO, we found that EPO therapy increased the preoperative Hb. The increased erythropoiesis that occurs early postoperatively has a direct effect on blood loss [24]. We found that the initial threshold Hb level of 12 g/dl for EPO implementation was too high in our practice.

The primary drawback of preoperative EPO therapy is its expense. However, the financial burden of EPO therapy must be weighed against the expenses of blood transfusions as well as the health and economic consequences of complications associated with postoperative anemia. Given that the costs and safety of EPO therapy are dose-dependent, we assert that our model is economical and safe. We used a lower Hb threshold, a lower dose, and a shorter duration of EPO therapy than reported in the literature [10]. In this study, most patients with preoperative Hb levels < l1 g/dl were treated with EPO therapy, while only a minority of patients with baseline Hb levels < 12 g/dl received EPO. The threshold we now consider for the use of EPO or EPO biosimilar medication is 11 g/dl.

Erythropoietin is a hormone produced by the kidneys, and its production is reduced in renal patients. Hence, EPO recombinant therapy is deemed to be more effective for patients with kidney issues. Notably, EPO therapy is considered safe when utilized at a low dose, particularly for individuals with renal problems. In the current series, more than 40% of ACD patients had a preoperative MDRD less than 60 ml/min/m^2^.

### Tranexamic acid

With the ubiquitous use of TXA and advancements in surgical techniques in recent years, the previous postoperative Hb drift of 3.8 g/dl (with a standard deviation of 1 g/dl) associated with total knee arthroplasty [20] has been mitigated to < 3 g/dl in modern TKA procedures. In the current study, a single infusion of TXA was given prior to the skin incision. No adverse effects were observed with TXA administration. Our approach involved a single adequately dosed shot to inhibit fibrinolysis during peak blood loss, without the need for repeated doses. Multiple trials have confirmed the efficacy of single-shot TXA compared to repeated-dose regimens [14, 25, 26]. The use of TXA plays a positive role in primary TKA, reducing the occurrence of hematomas [27], hidden blood loss, and the need for allogeneic blood transfusions. TXA reduces the transfusion rate from 23.3% to 9.1% in patients with renal impairment [28].

### Thromboprophylaxis

In the present series, all patients, except those with mechanical heart valves, received a 10 mg dose of direct-acting oral anticoagulant (DOAC) rivaroxaban once a day for a minimum of two weeks. We adopted rivaroxaban in 2009, as it reduces thrombotic events, bleeding risk, and drug monitoring requirements. No dose adjustment was implemented in relation to body weight or renal function. However, pauses were applied in cases of local or other nasal or urinary postoperative bleeding.

We did not use rivaroxaban in combination with other blood thinner agents to avoid provoking a marked prolongation of clotting time and to avoid increasing the risk of articular and periarticular bleeding and other complications related to excessive anticoagulation. Chronic anticoagulation was resumed once the risk of hemorrhage had mitigated |12.

### Strengths and limitations

One strength of the present study is that all data were collected prospectively as a part of regular clinical practice. The BI-7 data provided support for implementing EPO therapy. The efficiency and safety of the standardized PBM were evaluated over a long period (a decade) in a homogeneous study in terms of surgical technique and perioperative care protocols. A limitation of the study is that it only included a few patients with ACD. The group was too small to determine any differences in the rate of adverse drug reactions. All observational studies, i.e., studies without random group allocation of patients, are at risk of confounding due to unmeasured and/or unknown factors, which is another limitation of this study. The generalizability of our findings to other clinical settings needs to be determined. Further, given the monocentric nature of the current study and the fact that bleeding in orthopedic procedures varies between surgical teams, the reproducibility of our study needs to be confirmed by others. Another limitation is that the bleeding index is a confounded measure. The drop in Hb during the first week following TKA is influenced by several factors, including surgical and postoperative blood loss, hemodilution, transfusion (which is accounted for in the BI), and reduced erythropoiesis due to surgery-associated inflammation [29]. The effect of blood loss alone cannot be isolated, particularly not in the first few postoperative days. Nevertheless, in general, perioperative blood loss is the most significant factor.

Overall, this study shed light on clinical practice realities, provide clinical evidence, and support treatment recommendations. Observational studies have the potential to generate new hypotheses and identify specific patient groups, such as anemic patients, who may benefit significantly from a particular treatment. Thus, they contribute positively to our understanding of healthcare outcomes.

## Conclusion

In conclusion, transfusion for primary TKA was not needed with the application of the adjusted PBM in ACD patients. Pretransfusion testing (type and approximate screens) was not implemented because this practice appeared to be safe and cost-effective. ACD patients did not have an increased risk of bleeding or bleeding complications with the use of an adjusted surgical PBM. We now recommend using EPO at a baseline Hb level of 11 g/dl in ACD patients without nutritional anemia. However, this threshold should be validated in larger cohorts with a higher prevalence of ACD patients than in this trial.

## Data Availability

The datasets used and/or analyzed during the current study are available from the corresponding author on reasonable request.
